# Enhanced Lymph Node Detection in Colon Cancer Using Indocyanine Green Fluorescence: A Systematic Review of Studies from 2020 Onwards

**DOI:** 10.3390/jpm15020054

**Published:** 2025-01-29

**Authors:** Roxana Loriana Negrut, Adrian Cote, Bogdan Feder, Florian Dorel Bodog, Adrian Marius Maghiar

**Affiliations:** 1 Department of Medicine, Doctoral School of Biomedical Sciences, Faculty of Medicine and Pharmacy, University of Oradea, 410087 Oradea, Romania; popa.roxanaloriana@student.uoradea.ro (R.L.N.);; 2 County Clinical Emergency Hospital Bihor, 410087 Oradea, Romania; bogdanfeder@yahoo.com; 3 Department of Surgical Disciplines, Faculty of Medicine and Pharmacy, University of Oradea, 410073 Oradea, Romania

**Keywords:** colon cancer, colon neoplasm, colonic malignancy, surgery, ICG, indocyanine green, fluorescence, sentinel lymph node, lymphatic nodes, lymph node mapping

## Abstract

**Background:** Colon cancer is known as one of the most prevalent malignancies in the world. This well-known pathology requires accurate lymph node dissection to achieve effective staging and improved treatment outcomes. Indocyanine green fluorescence imaging has been used as a new technique for enhancing lymph node visualization during surgical intervention. The high rates of local recurrence in colon cancer patients require innovative methods to improve lymphatic mapping and lymph node dissection. This review evaluates the clinical utility and efficacy of ICG imaging in enhancing lymph node accuracy in colon cancer surgery. **Materials and methods:** A systematic search was conducted in October 2024 (last day of consulting the database was 16 November) across Web of Science, Scopus, and PubMed to identify studies published from 2020 onwards focusing on the use of indocyanine green in colon cancer surgeries. The search terms used were “indocyanine green”, “ICG”, “fluorescent imaging”, “near-infrared imaging”, “colon cancer”, “colorectal cancer”, “colon carcinoma”,” colon neoplasms”, “surgery”, “surgical procedure”, “surgical resection”, surgical precision”. The search followed PRISMA guidelines. The records underwent a two-phase independent screening process conducted by the authors, first based on the title and abstract, followed by full record evaluation. Articles were excluded following certain exclusion criteria: non-human studies; restricted access publications; other publication type than article (review, meta-analysis, questionnaire-based study, case report, etc.), studies focusing on other diseases or studies that focused on the surgical treatment of metastasis from colon cancer; foreign language (non-English); no data of interest for the current review; studies that focused on rectal cancer and that grouped rectal and colon cancer. Data extraction involved both quantitative and qualitative data, such as detection rates, sensitivity, specificity, and other surgical outcomes. Risk of bias was assessed using ROBINS-I, J Joanna Briggs Institute (JBI) Critical Appraisal Checklist, and the Newcastle–Ottawa Scale, depending on study type. The study was not preregistered in PROSPERO. However, to ensure methodological rigor and transparency, it was retrospectively registered in Open Science Framework (OSF). **Results:** From the 3300 records initially identified, 9 studies were included in this review. Detection rates varied from 55% to 100%, with the highest rate reported in robot-assisted surgeries. The studies showed an improved lymph node detection and lymphatic flow accuracy using ICG fluorescence. **Discussion:** ICG fluorescence demonstrated substantial benefits, improving staging accuracy and potentially reducing recurrence rates by guiding the lymphadenectomy. The variability observed in detection rates is largely attributed to differences in ICG administration, cancer stage, and surgical approaches. **Conclusions:** ICG-guided surgery for colon cancer represents a promising advancement, enhancing lymph node detection and staging accuracy. Large-scale randomized trials are essential to establish standardized protocols and validate the efficacy in improving surgical outcomes.

## 1. Introduction

Colon cancer is known as one of the most prevalent malignancies in the world and one of the leading causes of cancer-related mortality worldwide, necessitating continuous advancements in surgical techniques to improve patient outcomes. The key principles of oncologic surgery in colon cancer are accurate tissue dissection following anatomical embryological planes, regional lymphadenectomy, and ligation of the feeding artery [1]. Complete mesocolic excision combined with central vascular ligation has been standardized as the radical surgical approach, whether performed through laparotomy or laparoscopy [1,2]. Therefore, it is crucial to identify the lymphatic flow. Sentinel lymph node detection for colon cancer was first introduced in early 2000 for the purposes of identifying the primary tumor-draining lymph node and applying advanced histopathological techniques to enhance the accuracy of nodal staging [3,4,5]. Precision in identifying lymphatic tissues during surgery is critical as it influences the staging and treatment decisions.

Despite advancements, patients diagnosed and treated for colorectal cancer have a high local recurrence rate of 60% [6], primarily due to an incomplete resection rate of 30% [7], which is often caused by a positive tumor margin and inadequate mapping of proximal lymph nodes [4,8,9,10].

Accurate staging of colon cancer is essential to establish appropriate treatment to improve patient outcomes. Recent advancements introduced indocyanine green (ICG) fluorescence imaging to enhance visual identification of lymph nodes during surgery and to detect the sentinel lymph node (SLN).

ICG-guided surgery has been shown to be an effective technique for detection of metastatic lymph nodes following intravenous administration [9]. The utilization of ICG in surgical oncology, particularly in colon cancer, represents a significant shift towards precision medicine. This technique leverages the fluorescent properties of ICG to provide real-time, high-definition imaging of lymph nodes, which is crucial for effective lymphadenectomy. The adoption of such advanced imaging techniques is part of a broader trend towards adopting more targeted and minimally invasive approaches in cancer surgery, which aligns with current healthcare goals to maximize patient recovery while minimizing surgical risks.

This systematic review aims to synthesize recent findings on the clinical utility, efficacy, and feasibility of ICG fluorescence in enhancing lymph node detection and optimizing surgical outcomes in colon cancer patients. The included studies examine outcomes such as detection rates, upstaging rates, accuracy, sensitivity, and specificity, gathering insights into the benefits and limitations of using ICG guided surgical technique. Furthermore, understanding the variability in the application of ICG across different clinical settings and patient populations can elucidate factors that influence its effectiveness, thereby guiding standardized practices.

ICG fluorescence guided surgeries seem to be feasible and effective, with higher detection rates and improvements of lymph node dissection. The implications of this research extend beyond the immediate surgical techniques, influencing both long-term care strategies and potentially affecting survival rates among colon cancer patients.

## 2. Materials and Methods

### 2.1. Search Strategy

A systematic search of the existing literature was conducted in October 2024 (last day of consulting the database was 16 November 2024) focusing on the use of indocyanine green in colon cancer surgeries, using the databases Web of Science, PubMed, and Scopus. The review was conducted and adhered strictly to the Preferred Reporting Items for Systematic Reviews and Meta-Analyses (PRISMA) guidelines, which are designed to ensure transparency and completeness in reporting. The search terms used were “indocyanine green”, “ICG”, “fluorescent imaging”, “near-infrared imaging”, “colon cancer”, “colorectal cancer”, “colon carcinoma”,” colon neoplasms”, “surgery”, “surgical procedure”, “surgical resection”, surgical precision”. The search was carried out by using Boolean operators (AND, OR) in the search query and brackets were used to group search terms. Field-specific operators were not used; “All Fields” was used for the search. After the search, any other type of publication except “Article” was excluded by using the website filters. To provide a contemporary analysis, the publication period was filtered for articles published from January 2020 onwards. This timeframe was chosen to focus on the most current practices and innovations in the application of indocyanine green in colon cancer surgeries.

The databases searched included PubMed, Scopus, and Web of Science, which are recognized for their extensive coverage of medical and scientific publications.

Studies were included if they were original articles reporting the use of ICG in surgical settings for colon cancer that provided original data on outcomes such as detection rates, accuracy of lymph node mapping, or patient outcomes.

The literature review was conducted in accordance with the Preferred Reporting Items for Systematic Reviews and Meta-Analyses (PRISMA) guidelines [11,12]. The protocol was not preregistered.

### 2.2. Study Selection

The records were introduced to the Rayyan platform (Quatar Computing Research Institute) [13] for the removal of the duplicates and for the blind screening process.

Eligibility criteria:

Population: Patients diagnosed with colon cancer, undergoing open or laparoscopic or robotic surgery.

Intervention: Use of ICG fluorescence for lymph node detection.

Outcomes: Detection rates, sensitivity, specificity, staging accuracy, and adverse events.

Articles were excluded following certain exclusion criteria:

1. In vitro, cadaveric, or animal studies.
2. No full text available. Restricted access.
3. Other publication type (review, meta-analysis, questionnaire-based study, case report or case series, etc.).
4. Other diseases or studies that focused on the surgical treatment of metastasis from colon cancer.
5. Foreign language (not in English).
6. No data of interest for the current review.
7. Rectal cancer and studies that grouped rectal and colon cancer.

In selecting studies for inclusion in this review, strict criteria were applied to ensure the relevance and quality of data. Only the studies published from 2020 onwards, utilizing ICG in colon cancer surgeries, and reporting specific outcomes, such as detection rates and staging accuracy, were considered. Studies not in English, reviews, meta-analyses, and those involving non-human subjects were excluded to maintain a focus on recent, high-quality clinical data. The choice of these nine studies reflects a concentration on the latest advancements in surgical techniques that are both innovative and directly applicable to current clinical practices.

### 2.3. Data Extraction

Two reviewers independently extracted data using a defined template that included author, year of publication, country, study type, number of participants, patient demographics, ICG application techniques, lymph node detection metrics, surgical procedure and outcomes such as detection rates, sensitivity, specificity, complications, and oncological outcomes.

Discrepancies in data extraction were solved through group discussion including all five authors.

Primary outcomes are lymph node harvested, detection rates, diagnostic accuracy. Secondary outcomes involved changes in the intraoperative decision-making, adverse events, and feasibility of ICG use.

The method of synthesis involved both qualitative and quantitative approaches. Qualitative synthesis was conducted to explore the themes and patterns across the studies, while qualitative synthesis was limited due to the heterogeneity of the studies in terms of methodologies and outcomes reported.

### 2.4. Assessment

Each of the studies included in the systematic review was independently assessed for the risk of bias and applicability to ensure the integrity and applicability of the findings. This assessment was independently conducted by two of the authors to minimize any potential for bias in the evaluation process. The tools used were Risk of Bias in Non-randomized Studies of Interventions (ROBINS-I) [14], Joanna Briggs Institute (JBI) Critical Appraisal Checklist [15], and the Newcastle–Ottawa Scale (NOS) [16], depending on each study. For non-randomized studies, the ROBINS-I tool was used, which is specifically designed to evaluate bias in terms of confounding, selection of participants, classification of interventions, deviations from intended interventions, missing data, measurement of outcomes, and selection of the reported results. The ROBINS-I tool is comprehensive and allows for a detailed assessment of bias that might influence the validity of study conclusions.

Additionally, for studies that did not fit the criteria for assessment with ROBINS-I, the Joanna Briggs Institute (JBI) Critical Appraisal Checklist was used. This checklist is useful for a broad range of study designs, including randomized and quasi-randomized trial, providing a structured method to critically appraise the methodological quality of the studies. It focuses on various aspects of the study design, including the appropriateness of the statistical analysis, the reliability of the measurements, and the impact of study methods on the trustworthiness of the results.

For cohort studies, the Newcastle–Ottawa Scale (NOS) was utilized. This scale assessed three broad perspectives: the selection of the study groups, the comparability of the groups, and the ascertainment of either the exposure or outcome of interest for the case–control or cohort studies. The NOS helps in determining how well a study addressed potential confounding variables and biases in its design and methodology.

The decision to use these varied tools stems from their established validity in different research contexts, allowing for a tailored assessment approach that matches the specific designs of the studies included in the review. This multifaceted assessment strategy ensures a thorough understanding of the extent to which bias could have influenced the study outcomes, thereby strengthening the reliability of this systematic review’s conclusions.

Furthermore, it is important to note that the study was not initially registered with PROSPERO. However, recognizing the importance of transparency and reproducibility in research, we opted for a retrospective registration with the Open Science Framework (OSF)t. This registration, available at https://osf.io/cva2k (Accessed on 14 December 2024), provides a public record of our research plan and methodology, enhancing the credibility and allowing other researchers to understand, replicate, or build upon our research framework. This step, while not substituting for prospective registration, reflects our commitment to upholding rigorous scientific standards and enhancing the trustworthiness of our findings.

## 3. Results

### 3.1. Study Selection

A systematic literature search was performed by using three databases: Web of Science, PubMed, and Scopus. The search was carried out using Boolean operators (AND, OR) and brackets to group search terms and determine the order of operations in a search query. It is noted that only “Articles” were included, with any other type of publication (review, early access, abstract, proceeding paper, case report/series, etc.) being excluded through the website filters.

**Figure 1:**
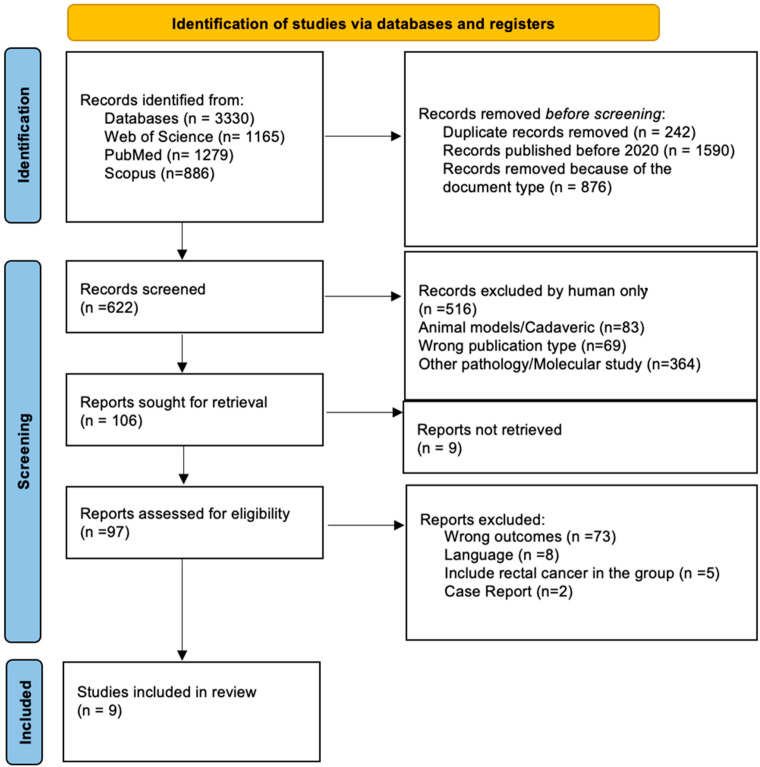
PRISMA flow diagram.

A total of 3300 records were identified, 1165 from Web of Science, 886 from Scopus, and 1279 from PubMed. Records published before 2020 (n = 1590) were excluded by using filters. A total of 876 records were removed due to the publication type in the same manner. Several duplicates (n = 242) were removed by automation tools. After the automatization tools removal, 622 articles remained for the screening process. All records were imported to the Rayyan platform. Three independent reviewers (R.L.N., A.C., B.F.) screened each record for title and abstract. Disagreements were solved by group discussion among all five authors. After the first round of screening, 516 records were excluded. A total of 97 studies were assessed for eligibility based on full-text screening. Each reviewer screened about 1/3 of the articles. Uncertainty about eligibility was solved by discussion among all the authors. Following a thorough selection process, nine studies were selected for this systematic review. The PRISMA guidelines [11,12] were followed in the selection process and are depicted in Figure 1.

### 3.2. Risk of Bias

The risk of bias was assessed by two authors (R.L.N. and A.C.) independently, followed by a group discussion to address the discrepancies. Disagreements were solved through discussion with all of the authors. To evaluate the risk of bias for the selected studies, different assessment tools were used based on the design of each study. For non-randomized prospective intervention studies, ROBINS-I was used for the articles written by Daan J. Sikkenk et al. [17], Zeeshan Ahmed et al. [18], Weiyang Lin et al. [19], Bianca Maria Sollazzo et al. [20], Gyung Mo Son et al. [21], Gyung Mo Son et al. [22], and Hiromitsu Kinoshita et al. [23]. This tool was selected due to the non-randomized intervention, requiring assessment across several domains, including confounding, selection, and deviations from intended intervention. The study published by Hokuto Ushijima et al. [24] is a single-arm observational study; the JBI Critical Appraisal Checklist thus being more suitable for evaluating this study. A retrospective study conducted by Xiaochuang Feng et al. [25] was assessed by using the NOS. By using these tools, the risk of bias was assessed to align with the design of each study, ensuring an accurate evaluation. The assessed risk of bias is presented in Table 1.

For the NOS, each study was evaluated across three domains: selection, comparability and outcome. Each domain was allocated a number of stars; a total of 7–9 stars indicating low risk of bias, 4–6 indicating moderate risk, less than 4 stars suggesting high risk of bias [16]. For ROBINS-I, seven domains were evaluated: confounding, selection of participants, classification of interventions, deviations from intended interventions, missing data, outcome measurement, and selection of reported results. Based on ratings for each domain, the overall risk was categorized as low, moderate or high [14]. For the JBI Critical Appraisal Checklist, each study was assessed for ten criteria, the number of “yes” responses was used to categorize the risk. The number of “yes” responses between 7 and 10 indicated low risk, 4–6 suggested moderate risk, and under 4 indicated high risk [26].

### 3.3. Studies Characteristics

From the nine studies included in this review, two were conducted in South Korea, two in China, two in Japan, one in India, one in The Netherland, and one in Italy. All studies were published between 2020 and 2024. All studies used ICG as a tracer during surgical procedures (robot-assisted, laparoscopic and open surgeries). One study additionally employed Nanocarbon as tracer [19].

The surgical approach primarily used laparoscopic techniques, with one study using a robot-assisted approach [17] and one study using laparoscopic and open resection [18]. The number of participants ranged from 10 [17] to 291 [22].

The inclusion criteria varied, encompassing different cancer stages, from early cancer to advanced cases, with several studies focusing on right-sided colon cancer. Each study characteristics are presented in Table 2.

ICG was used to enhance detection rates of SLNs and lymphatic mapping across a variety of surgical approaches. The detection rates ranged from 55% to 100%, with most studies reporting improved outcomes with ICG use, such as increased detection rates of lymph nodes and better mapping accuracy. Daan J. Sikkenk at al. [17] reported a 100% detection rate of SLNs with no complications. Only the study conducted by Zeeshan Ahmed et al. [18] reported the finding of four false negative SLNs. The lack of complications indicates the safety of ICG-guided surgeries. Sensitivity was reported as high in all studies, particularly in early-stage cancers. Table 3 shows a comprehensive summary of the interventions and outcomes.

The study by Daan J. Sikkenk et al. [17] investigated robot-assisted sentinel lymph node identification in patients diagnosed with early-stage colon cancer, obtaining detection rates of 100% with a mean of 2.3 SLNs identified per patient. Sensitivity was not explicitly reported, but there were no false negatives observed. Upstaging was noted in two patients who had two nodes identified as positive. ICG was administered submucosally during colonoscopies. Detection occurred within an average of 6 min.

Zeeshan Ahmed et al. [18] used a cohort of 48 patients diagnosed with T1-3 colon cancer. They achieved a detection rate of 93.75% for SLNs. The sensitivity was 77.77%, with a higher sensitivity of 90% observed for T1-2 tumors. The upstaging rate was 10%. ICG was administered submucosal for laparoscopic procedures and subserosal for open surgeries. They achieved an average detection time of 8.2 min.

The study conducted by Hokuto Ushijima et al. [24] on 57 patients who underwent ICG imaging for lymphatic flow detection during laparoscopic surgery for colon cancer found that the detection rates varied by stage, with an overall visualization rate of 75.4%. Sensitivity and specificity were not reported explicitly. Upstaging information was also not provided. ICG was administered submucosally during colonoscopy.

Weiyang Lin et al. [19] compared ICG fluorescence with nanocarbon staining in 30 patients with colon cancer. ICG imaging demonstrated an area under a curve of 0.931, indicating a high sensitivity. There was no upstaging information. ICG was administered submucosally, 16–24 h before surgery and during colonoscopy. They observed that fluorescent nodes displayed weaker signals in metastatic lymph nodes that in non-metastatic.

Bianca Maria Sollazzo et al. [20] conducted a study on 20 patients undergoing laparoscopic right hemicolectomy with D3 lymphadenectomy. ICG-FI identified lymph nodes in 55% of cases. Upstaging occurred in eight patients based on ICG findings. Sensitivity and specificity were not reported. ICG was injected intraoperatively into the submucosa near the tumor. They reported intraoperative changes made based on lymphatic flow visualization.

Gyung Mo Son et al. [21] evaluated 218 patients undergoing right hemicolectomy. The use of ICG for lymph node mapping improved detection of D3 lymph node metastasis. Sensitivity and specificity were improved for advanced cases compared to conventional methods. Upstaging was facilitated by better identification of metastatic nodes. ICG was administered by endoscopy in the submucosa.

In the study by Xiaochuang Feng et al. [25] on lymph node distribution in 143 patients with right-sided colon cancer, ICG showed a metastasis probability of 2.1% in central lymph nodes. Sensitivity and specificity were not explicitly provided. Upstaging was not included. ICG was injected into the submucosa during a preoperative colonoscopy.

Gyung Mo Son et al. [22] conducted a case–control study that included 291 patients undergoing laparoscopic right hemicolectomy. ICG imaging increased the harvested D3 lymph node count by 50% and doubled metastatic node detection in stage III colon cancer. The administration of ICG was made through endoscopy by submucosal injection. Upstaging occurred in patients with previously undetected metastasis.

Hiromitsu Kinoshita et al. [23] conducted a prospective study on 56 patients undergoing laparoscopic surgery. They observed ICG fluorescence lymphatic flow in 76.8% cases within 30–60 min of injection. Two patients had metastasis outside the fluorescent zones. Data regarding upstaging was not provided. ICG was injected subserosally at multiple sites around the tumor during surgery. Lymphatic node metastasis was observed in 14 cases. Fluorescence was detected beyond the standard dissection range in 20.9% of cases, additional resection areas being included.

The detection rate of SLNs using ICG ranged from 75.4% to 100%. Daan J. Sikkenk et al. [17] and Zeeshan Ahmed et al. [18] reported rates close to 100%, indicating that ICG is highly effective for the identification of SLNs during colon cancer surgery.

Sensitivity was reported in four out of nine studies, ranging from 62.9% to 90%. Zeeshan Ahmed et al. [18] reported a sensitivity of 77.77% with a specificity reaching 100%, highlighting ICG’s accuracy in detecting metastatic lymph nodes.

Zeeshan Ahmed et al. [18] reported upstaging rates of 10% after sentinel lymph node analysis. Gyung Mo Son et al. [21] reported 6.8% and Daan J. Sikkenk at al. [17] 0%. Other studies did not explicitly measure upstaging.

ICG was commonly administered submucosally during colonoscopy or subserosally during surgery. Timing differed, with some studies injecting ICG immediately before surgery, while others administering it 16–24 h in advance.

## 4. Discussion

ICG is a clinically approved tricarbocyanine iodide dye with excellent solubility in water and an amphiphilic nature, in addition to a strong affinity for plasma proteins. When subjected to near-infrared (NIR) light, it emits fluorescence, with excitation and emission peaks at 780 nm and 820 nm [27,28,29]. It is metabolized in the liver and excreted through bile without being absorbed in the intestine, having low toxicity, which facilitates a fast bloodstream circulation and accumulation in lymphatic pathways and regional lymphatic nodes [29,30,31,32].

The role of ICG in colon cancer surgery was found to be a transformative approach to improve staging accuracy and surgical outcomes.

The timeframe for selecting studies, limited to those published after 2020, was chosen to focus on the latest advancements in the use of ICG in surgical applications, reflecting current technologies and practices. This approach ensures that the review reflects the most up-to-date data, given the rapid evolution in surgical techniques and imaging technologies.

The implementation of ICG fluorescence in SLN identification has shown its potential in enhancing lymphatic mapping.

Various authors suggested different protocols for ICG dosage, methods of administration, and timing, but a universally accepted standard has not been established yet [33]. The dose concentration ranges from a very low volume of 0.2 mL (concentration 0.25 mg/mL) [4,34] to large volumes of 2–5 mL (concentration of 5 mg/mL) [35,36,37,38]. This variability is due to different approaches used for ICG injection. The injection technique involves submucosal injections by endoscopic intervention [39,40,41,42,43], subserosal injections during surgery [35,36,37,44], and a combination of these techniques or intravenous injection [4].

A study published by Daan J. Sikkenk et al. [17] involving early-stage colon cancer showed that robot-assisted ICG-guided surgery achieved a 100% detection rate, identifying lymph nodes even with a 1 mm diameter. The robotic platform provided enhanced precision with a submucosal injection of ICG that allowed efficient localization of SLNs within an average time of 30.5 min. A prospective study by Zeeshan Ahmed et al. [18] conducted on 48 Indian patients recorded a 93.75% detection rate for SLNs in ICG guided surgery by laparoscopic or open approach, with high sensitivity particularly for T1-T2 tumors. These findings show the safety and feasibility of ICG lymphatic mapping.

A study conducted by Weiyang Lin et al. [19] compared ICG fluorescence with nanocarbon dyes. The findings demonstrated a stronger correlation for ICG, achieving an area under curve of 0.931 for lymph node metastasis detection.

The visualization of lymphatic flow using ICG allows surgeons to adapt the extension of lymphadenectomy intraoperatively. Bianca Maria Sollazzo et al. [20] found a 55% incidence of lymph node metastasis and Hiromitsu Kinoshita et al. [23] showed that in 20.9% of cases, metastatic lymph nodes were located outside the standard dissection range, requiring surgical adjustments. These results highlight the necessity of real-time imaging that enhances surgical precision.

Studies suggest that ICG effectiveness varies with the tumoral stage. Daan J. Sikkenk et al. [17] observed high sensitivity and no false negatives in SLN detection for early-stage tumors. Also, Zeeshan Ahmed et al. [18] reported a 90% detection rate for early-stage tumors. These studies support ICG efficacy in intact lymphatic systems. For advanced tumoral stages, Hokuto Ushijima et al. [24] highlight the limitations of ICG due to disrupted lymphatic pathways, although still showed that ICG provides valuable identification of lymphatic spread beyond conventional dissection ranges. Gyung Mo Son et al. [22] demonstrated that the metastatic lymph node detection rate was doubled with fluorescence use, showing significantly enhanced lymph node yield during D3 dissection.

Hiromitsu Kinoshita et al. [23] and Hokuto Ushijima et al. [24] mention that there are challenges such as inconsistent fluorescence intensity in advanced cancer that depend on injection technique, drowning the conclusion that addressing technical limitations and standardized protocols will lead to broader adoption.

ICG fluorescence is a reliable and safe method for assessing lymphatic flow and SLN detection in colon cancer, even though its overall sensitivity, specificity, and accuracy fluctuate depending on the technical variables [45].

In reviewing the selected studies, we recognize the significance of addressing the potential biases that may impact the generalizability and accuracy of our findings. Detection bias could have occurred, given that different studies employed varying thresholds and techniques for identifying positive lymph node detections. To mitigate these concerns, future research should adopt a more rigorous randomized controlled trial design, which can help minimize selection and detection biases. Additionally, including larger and more diverse patient populations in studies cand enhance the robustness of the data and ensure that the findings are applicable across different demographic groups.

The challenges posed by the heterogeneity of the studies included in this systematic review are recognized. The diversity in study designs, patient populations, and methodologies across the studies significantly impacts the comparability and synthesis of the findings. The implications of this heterogeneity were examined for the review’s conclusion. First, the studies are categorized based on their design and methodology to assess the impact of these factors on the reported outcomes. This categorization helped in identifying patterns and differences that may arise. Second, the variability in patient populations across different studies might affect the generalizability of the findings. For example, studies conducted in different geographical locations or healthcare settings may report varied outcomes due to the systemic differences. The methodologies employed in these studies were discussed, highlighting the differences in technique, such as the use of ICG dosage and imaging equipment. In light of this heterogeneity, the conclusions were carefully drawn to avoid overgeneralization.

The findings from this systematic review suggest that ICG fluorescence is a valuable tool for lymphadenectomy in colon cancer, improving the dissection. The high detection rate observed support its feasibility, while the improvements in accuracy and reduced rates of false negatives indicate the potential to enhance surgical outcomes. The outcomes can be influenced by factors such as surgeon experience, injection technique, and tumor location.

The findings of this review support recent advancements in the utilization on ICG during colon cancer surgeries, as reflected in the included studies. However, our review also highlights variability in detection rates that may stem from different surgical techniques and ICG administration protocols. This discrepancy underscores the need for standardized practices, which are critical for optimizing clinical outcomes.

While the selected studies provide valuable insights, they are not without limitations. The potential bias due to the non-randomized design of most included studies could affect the generalizability of the findings. The variations in study methodologies may contribute to the discrepancies observed in lymph node detection rates. Acknowledging these limitations is crucial for interpreting the results accurately and applying them in a clinical context.

Future research should focus on large-scale randomized trials to confirm these benefits and help to establish standardized protocols for ICG use in colon cancer surgeries. The optimization of the protocols should be made by standardizing ICG concentration, timing, and injection techniques. The combination of ICG fluorescence with artificial intelligence and advance imaging modalities in the future could enhance real-time lymph node mapping, as suggested by Weiyang Lin et al. [19].

Studies presented in the literature mention that ICG fluorescence imaging for lymphatic flow observation has limitations particularly in patients with bulky lymph node metastasis, where the evaluation is hindered by the obstruction and alteration of the lymphatic flow [46,47,48]. Lymphatic flow can extend centrally, reaching to areas that may lead to an extensive dissection [48]. ICG lymphatic flow mapping is useful in defining the extension of lymph node dissection for patients with early-stage colon cancer, with predilection to those located in the hepatic and splenic flexure [24].

## 5. Conclusions

ICG-guided surgery seems to be a promising advancement in lymphadenectomy for colon cancer by enhancing sentinel lymph node detection and lymphatic mapping. The studies analyzed indicate high detection rates and improved staging accuracy with ICG, making it a valuable addition to surgical practice.

However, additional studies are necessary to standardize the methodology and further evaluate the long-term outcomes. Meta-analysis could not be performed due to the heterogeneity of the studies. Another limitation of this review is that it was not preregistered in a systematic review registry, such as PROSPERO. However, to address this limitation, we retrospectively registered the review in the Open Science Framework (OSF). This retrospective registration ensures that our methodology and objectives are openly documented, providing transparency despite the lack of prospective registration.

Future research should focus on large-scale randomized trials to validate this technique compared to traditional methods and to establish standardized protocols for ICG use in colon cancer.

The main limitations of this study are the heterogeneity of the included studies resulting in the inability to perform a meta-analysis, the inconsistent outcome reporting, and the small sample sizes in several studies. Another limitation of the review is the exclusion of non-English language studies. While this decision was made due to the practical constraints of translation resources, it may have restricted the scope of our analysis. Including non-English studies could potentially enrich the findings. Future systematic reviews could consider incorporating a broader linguistic range to capture valuable insights from studies published in other major languages.

Despite these limitations, the fact that the detection rates are consistent and that all articles show improvement in diagnostic accuracy, we can conclude that incorporating ICG fluorescence into routine practice could enhance surgical precision and staging accuracy for colon cancer cases.

The incorporation of ICG-guided surgery into routine colon cancer surgery has the potential to set a new standard for precision oncology by improving staging accuracy and optimizing lymphadenectomy.

In conclusion, this systematic review corroborates the potential of ICG in improving surgical outcomes in colon cancer treatment. By consolidating current evidence and highlighting areas needing further investigation, this review serves as a call to action for continued research and development in this field. Emphasizing standardized methodologies will be crucial for advancing the practical application of ICG guided techniques in clinical settings, ensuring that all patients benefit from the latest surgical innovations.

## Figures and Tables

**Table 1:** Risk of bias assessment for each study.

Article	Study Type	Assessment Tool	Bias Domains Assessed	Overall Risk
Daan J. Sikkenk et al. [17]	Prospective Pilot Study	ROBINS-I	Confounding, Selection, Classification, Deviations, Missing Data, Outcome Measurement, Reported Results	Moderate
Zeeshan Ahmed et al. [18]	Prospective Cohort Study	ROBINS-I	Confounding, Selection, Classification, Deviations, Missing Data, Outcome Measurement, Reported Results	Moderate
Hokuto Ushijima et al. [24]	Observational Study	JBI Critical Appraisal Checklist for Case Series	Inclusion Criteria, Outcome Measurement, Consecutive Participants, Demographics, Statistical Analysis	Moderate
Weiyang Lin et al. [19]	Prospective Study	ROBINS-I	Confounding, Selection, Classification, Deviations, Missing Data, Outcome Measurement, Reported Results	Moderate
Bianca Maria Sollazzo et al. [20]	Non-Randomized Prospective Pilot Study	ROBINS-I	Confounding, Selection, Classification, Deviations, Missing Data, Outcome Measurement, Reported Results	Moderate
Gyung Mo Son et al. [21]	Non-Randomized Cohort Study	ROBINS-I	Confounding, Selection, Classification, Deviations, Missing Data, Outcome Measurement, Reported Results	Moderate
Xiaochuang Feng et al. [25]	Retrospective Study	NOS	Selection, Comparability, Outcome	Moderate
Gyung Mo Son et al. [22]	Prospective Case–Control Study	ROBINS-I	Confounding, Selection, Classification, Deviations, Missing Data, Outcome Measurement, Reported Results	Moderate
Hiromitsu Kinoshita et al. [23]	Prospective Study	ROBINS-I	Confounding, Selection, Classification, Deviations, Missing Data, Outcome Measurement, Reported Results	Moderate

**Table 2:** Study characteristics.

Article	Year	Country	Study Design	Surgical Intervention	Tracer	Number of Participants	Criteria
Daan J. Sikkenk et al. [17]	2023	The Netherland	Prospective	Robot-assisted	ICG	10	cT1-2N0M0
Zeeshan Ahmed et al. [18]	2023	India	Prospective cohort	Laparoscopic/ Open	ICG	48	T1-4aN0-2b
Hokuto Ushijima et al. [24]	2020	Japan	Prospective cohort	Laparoscopic	ICG	57	Any stage
Weiyang Lin et al. [19]	2024	China	Prospective	Not mentioned	ICG and Nanocarbon	30	Any stage
Bianca Maria Sollazzo et al. [20]	2020	Italy	Prospective	Laparoscopic	ICG	20	Any stage Right-sided colon cancer
Gyung Mo Son et al. [21]	2024	South Korea	Unclear	Laparoscopic	ICG	218	Any stage Right-sided colon cancer
Xiaochuang Feng et al. [25]	2021	China	Retrospective	Laparoscopic	ICG	143	Any stage Right-sided colon cancer
Gyung Mo Son et al. [22]	2023	South Korea	Prospective	Laparoscopic	ICG	291	Any stage Right-sided colon cancer
Hiromitsu Kinoshita et al. [23]	2023	Japan	Prospective	Laparoscopic	ICG	56	Any stage

**Table 3:** Comprehensive summary of interventions and outcomes.

Article	Intervention Description	Surgical Technique	Additional Notes	Detection Rate	Sensitivity	Complications
Daan J. Sikkenk et al. [17]	Robot assisted SLNi with submucosal ICG	Segmental colectomy	SLNs ultra staged with IHC	100% SLN detection	High	None
Zeeshan Ahmed et al. [18]	Colonoscopic ICG injection during surgery	Laparoscopic/open resection	Separate protocols for laparoscopic and open injections	93.75% SLN detection	77.77%; higher for T1-2	Four false negative SLNs
Hokuto Ushijima et al. [24]	Lymphatic flow visualization with ICG imaging	Laparoscopic resections	Real-time imaging used to guide resection	A 75.4% lymphatic flow visualization	Higher in early stage cancer	None
Weiyang Lin et al. [19]	Comparison of ICG vs. nanocarbon	Standard colon surgery	Dual tracers analyzed for node detection	Varied (ICG superior to nanocarbon)	ICG Strong correlation with metastasis	None
Bianca Maria Sollazzo et al. [20]	Fluorescence guided D3 lymphadenectomy	CME with CVL	Intraoperative changes made based on lymphatic flow visualization	A 55% higher incidence of lymph node metastasis	Improved compared to non ICG	None
Gyung Mo Son et al. [21]	FLNM using ICS for real-time visualization	Laparoscopic right colectomy	FLNM performed after endoscopic submucosal ICG injection	Improved detection of D3 nodes	High for D3 LN staging	None
Xiaochuang Feng et al. [25]	Preoperative ICG tattooing	SMA oriented colectomy	Mapping includes anterior/posterior to SMA lymph nodes	81.5% mapping of SMA-related lymph node	Variable	None
Gyung Mo Son et al. [22]	FLNM for improved DT dissection	Laparoscopic right colectomy	Enhanced oncological dissection using ICG	50% increase in harvested D3 nodes	High for metastatic nodes	None
Hiromitsu Kinoshita et al. [23]	Real-time ICG	CME with CVL	Lymphatic flow patterns to optimize dissection	20.9% cases showed ICG beyond standard dissection	High	None

## Data Availability

The datasets during and/or analyzed during the current study are available from the first and corresponding author on reasonable request.
